# Supplementary material for: Safety Evaluation of Tadalafil Treatment for Fetuses with Early-Onset Growth Restriction (TADAFER): Results from the Phase II Trial
Source: J Clin Med. 2019 Jun 15;8(6):856. doi: 10.3390/jcm8060856 (PMC6617029; doi:10.3390/jcm8060856)
Supplement: Supplementary file 1 [file jcm-08-00856-s001.zip › Supplemental File 1 JCM.pdf]

Supplemental File 1

The list of facilities that participated in the TADAFER study

Mie University Hospital, Showa University Hospital, Nagoya University Hospital, Osaka University Hospital, Sapporo City General Hospital, Dokkyo Medical University Hospital, Toho University Omori Medical Center, Tokyo Metropolitan Tama Medical Center, Jikei University Hospital, Yokohama City University Medical Center, Shinshu University Hospital, Kainan Hospital, Fukui University Hospital, Municipal Yokkaichi Hospital, Mie Chuo Medical Center, Ise Red Cross Hospital, Mie Prefectural General Medical Center, Shiga University of Medical Science hospital, Kyoto University Hospital, National Hospital Organization Saga Hospital, Nagasaki Medical Center, University of the Ryukyus Hospital.
