# Supplementary material for: Safety Evaluation of Tadalafil Treatment for Fetuses with Early-Onset Growth Restriction (TADAFER): Results from the Phase II Trial
Source: J Clin Med. 2019 Jun 15;8(6):856. doi: 10.3390/jcm8060856 (PMC6617029; doi:10.3390/jcm8060856)
Supplement: Supplementary file 1 [file jcm-08-00856-s001.zip › Supplemental File 2 JCM.pdf]

All cases

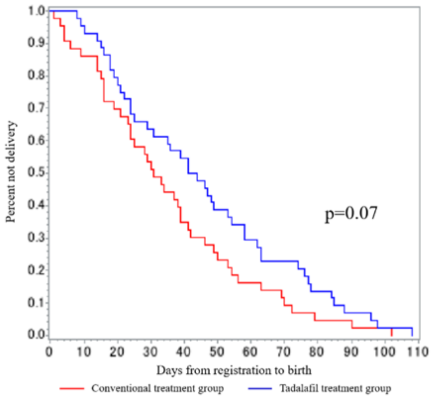

GA<32 weeks

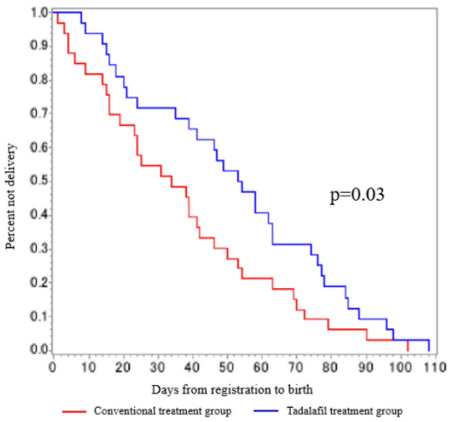

GA≧32 weeks

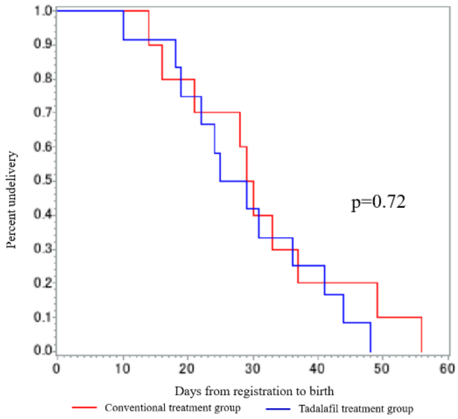

GA<30 weeks

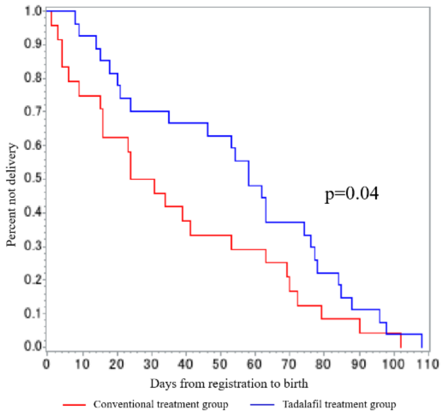

GA≧30 weeks

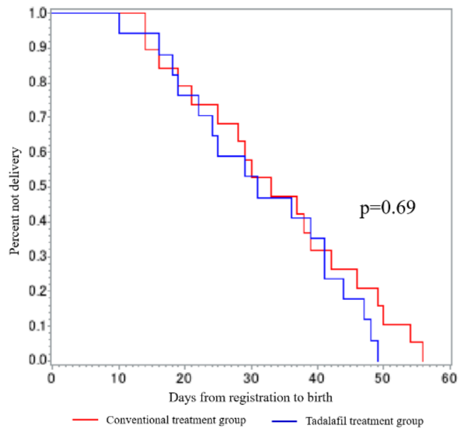

GA<28 weeks

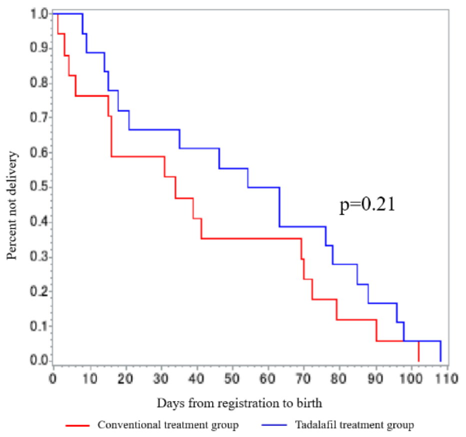

GA≧28 weeks

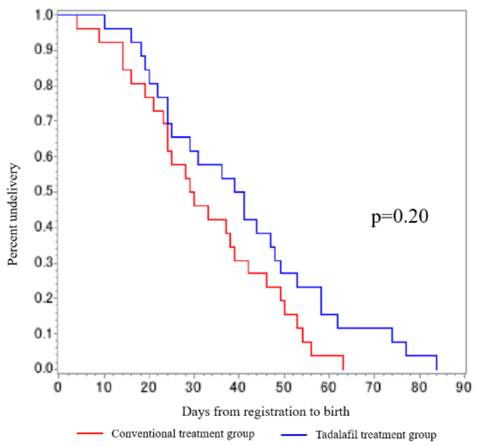

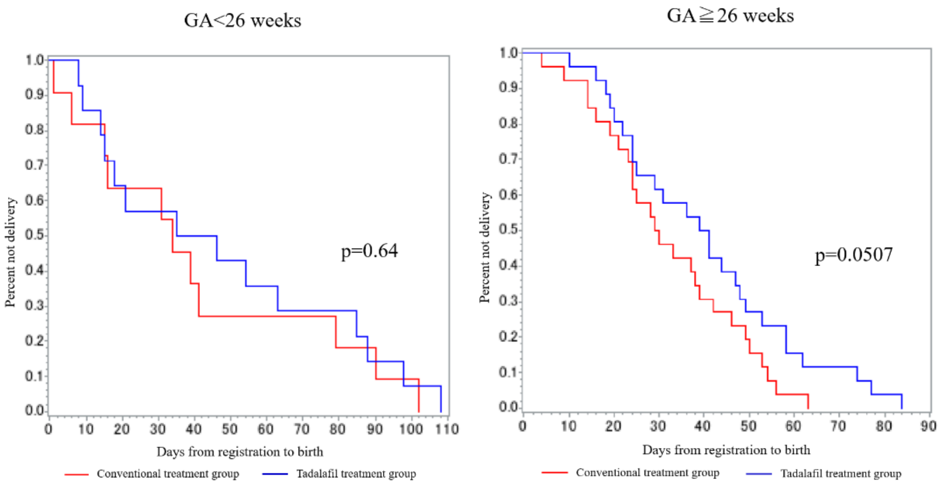

Figure S1. Prolongation of GA (post hoc analysis: Kaplan–Meier curves).

Table S1. Prolongation of GA (post hoc analysis: table).

	Tadalafil Treatment Group (*n* = 44)	Conventional Treatment Group (*n* = 43)	*P* Value
Prolongation of gestational age (days)			
All cases	46.0(27.3)	35.5(24.3)	0.06
<32 gestational weeks	52.4(28.9)	36.8(26.8)	0.03
≥32 gestational weeks	28.9(11.5)	31.3(13.4)	0.66
<30 gestational weeks	55.0(30.4)	37.0(30.5)	0.04
≥30 gestational weeks	31.7(12.4)	33.7(13.5)	0.65
<28 gestational weeks	54.2(34.3)	40.5(33.4)	0.24
≥28 gestational weeks	40.3(20.1)	32.3(15.8)	0.12
<26 gestational weeks	47.3(35.6)	41.3(34.4)	0.67
≥26 gestational weeks	45.4(23.2)	33.6(20.1)	0.04

Data are mean (standard deviation). Prolongation of GA divided according to the GA at the time of registration represented by Kaplan–Meier curves and table (post hoc analysis). The Prolongation of GA was defined as days from the first day of protocol-defined treatment to birth. Figure A1 shows the prolongation of GA in Kaplan–Meier curves for each GA at treatment started (<32 weeks, ≥32 weeks, <30 weeks, ≥30 weeks, <28 weeks, ≥28 weeks, <26 weeks, ≥26 weeks), compared binary data across two groups using generalized Wilcoxon test. Table S1 shows comparison of two groups by unpaired *t* test.

Table S2. GA at birth and birth weight divided by GA at registration (post hoc analysis).

	**Tadalafil Treatment Group (*n* = 40)**	**Conventional Treatmnent Group (*n* = 39)**	***P* value**
GA at birth			
<32 gestational weeks	37.00(31.50–37.86)	34.14(29.71–37.00)	0.16
≥32 gestational weeks	37.07(35.72–38.14)	37.15(36.00–39.14)	0.58
<30 gestational weeks	37.07(30.86–37.86)	31.50(28.93–36.15)	0.048
≥30 gestational weeks	37.00(35.72–37.86)	37.29(35.71–38.43)	0.72
<28 gestational weeks	35.93(28.79–37.86)	29.71(28.14–36.29)	0.25
≥28 gestational weeks	37.07(35.72–37.86)	36.57(32.71–37.71)	0.36
Birth weight			
<32 gestational weeks	1523(663)	1380(687)	0.43
≥32 gestational weeks	1909(388)	2035(568)	0.55
<30 gestational weeks	1470(678)	1185(644)	0.16
≥30 gestational weeks	1891(408)	1930(578)	0.82
<28 gestational weeks	1301(621)	1190(724)	0.66
≥28 gestational weeks	1864(509)	1727(649)	0.41

Data are median (interquartile range) or mean (standard deviation). The analysis of GA at birth and birth weight divided according to GA at registration as post hoc analysis are shown. When limited to cases registered at <30 weeks GA, there were significant differences in GA at birth between the tadalafil and conventional treatment groups.

Table S3. Doppler study analysis of maternal or fetal vessels divided according to gestational age at registration (post hoc analysis).

	Tadalafil Treatment Group (*n* = 40)	Conventional Treatment Group (*n* = 39)	*P* Value
Umbilical artery			
<32 gestational weeks			
PI 1 week from the start of treatment	1.00(0.91–1.32)	1.17(0.93–1.51)	0.26
PI 2 weeks from the start of treatment	1.08(0.93–1.28)	1.05(0.85–1.32)	0.90
PI 3 weeks from the start of treatment	0.96(0.83–1.30)	1.01(0.81–1.30)	0.82
≥32 gestational weeks			
PI 1 week from the start of treatment	0.89(0.80–1.05)	1.00(0.95–1.06)	0.30
PI 2 weeks from the start of treatment	1.11(0.76–1.37)	1.00(0.92–1.10)	0.74
PI 3 weeks from the start of treatment	0.78(0.71–1.06)	0.96(0.89–1.07)	0.16
Middle cerebral artery			
<32 gestational weeks			
PI 1 week from the start of treatment	1.67(1.39–2.08)	1.71(1.46–2.23)	0.56
PI 2 weeks from the start of treatment	1.77(1.37–2.10)	1.56(1.31–1.92)	0.46
PI 3 weeks from the start of treatment	1.89(1.53–2.25)	1.69(1.29–2.19)	0.33
≥32 gestational weeks			
PI 1 week from the start of treatment	1.61(1.31–2.04)	1.59(1.41–1.74)	0.97
PI 2 weeks from the start of treatment	1.61(1.24–1.63)	1.67(1.31–1.75)	0.50
PI 3 weeks from the start of treatment	1.51(1.15–1.57)	1.62(1.46–1.98)	0.16
Uterine artery			
<32 gestational weeks			
PI 1 week from the start of treatment	0.98(0.77–1.54)	0.77(0.67–1.10)	0.11
PI 2 weeks from the start of treatment	1.08(0.83–1.47)	0.94(0.73–1.35)	0.42
PI 3 weeks from the start of treatment	0.80(0.76–1.18)	1.08(0.65–1.57)	0.51
≥32 gestational weeks			
PI 1 week from the start of treatment	0.88(0.70–1.05)	0.93(0.76–1.59)	0.46
PI 2 weeks from the start of treatment	0.84(0.69–1.28)	0.94(0.75–1.03)	0.54
PI 3 weeks from the start of treatment	1.17(0.63–1.23)	0.72(0.43–1.39)	0.37

Data are median (interquartile range). The analysis of doppler study after the start of treatment divided according to GA at registration was shown as post hoc analysis. No significant differences were found between two groups.
